# Elucidating Drug-Enzyme Interactions and Their Structural Basis for Improving the Affinity and Potency of Isoniazid and Its Derivatives Based on Computer Modeling Approaches 

**DOI:** 10.3390/molecules15042791

**Published:** 2010-04-16

**Authors:** Auradee Punkvang, Patchreenart Saparpakorn, Supa Hannongbua, Peter Wolschann, Pornpan Pungpo

**Affiliations:** 1 Department of Chemistry, Ubon Ratchathani University, 85 Sthollmark Rd., Warinchamrap, Ubonratchathani, 34190, Thailand; 2 Department of Chemistry, Kasetsart University, Chatuchak, Bangkok, 10900, Thailand; 3 Institute for Theoretical Chemistry, University of Vienna, A-1090 Vienna, Austria

**Keywords:** isoniazid, CoMFA, CoMSIA, docking, quantum chemical calculations

## Abstract

The enoyl-ACP reductase enzyme (InhA) from *M. tuberculosis* is recognized as the primary target of isoniazid (INH), a first-line antibiotic for tuberculosis treatment. To identify the specific interactions of INH-NAD adduct and its derivative adducts in InhA binding pocket, molecular docking calculations and quantum chemical calculations were performed on a set of INH derivative adducts. Reliable binding modes of INH derivative adducts in the InhA pocket were established using the Autodock 3.05 program, which shows a good ability to reproduce the X-ray bound conformation with rmsd of less than 1.0 Å. The interaction energies of the INH-NAD adduct and its derivative adducts with individual amino acids in the InhA binding pocket were computed based on quantum chemical calculations at the MP2/6-31G (d) level. The molecular docking and quantum chemical calculation results reveal that hydrogen bond interactions are the main interactions for adduct binding. To clearly delineate the linear relationship between structure and activity of these adducts, CoMFA and CoMSIA models were set up based on molecular docking alignment. The resulting CoMFA and CoMSIA models are in conformity with the best statistical qualities, in which r^2^_cv_ is 0.67 and 0.74, respectively. Structural requirements of isoniazid derivatives that can be incorporated into the isoniazid framework to improve the activity have been identified through CoMFA and CoMSIA steric and electrostatic contour maps. The integrated results from structure-based, ligand-based design approaches and quantum chemical calculations provide useful structural information facilitating the design of new and more potentially effective antitubercular agents as follow: the R substituents of isoniazid derivatives should contain a large plane and both sides of the plane should contain an electropositive group. Moreover, the steric and electrostatic fields of the 4-pyridyl ring are optimal for greater potency.

## 1. Introduction

Tuberculosis (TB) caused by *Mycobacterium tuberculosis* (*M. tuberculosis*) still remains a major cause of illness and death worldwide, especially in Asia and Africa. Globally, 9.2 million new cases and 1.7 million deaths from TB occurred in 2006. The number of new cases was still increasing between 2005 and 2006, from 9.1 to 9.2 million (an increase of 0.6%) [1]. Antibiotics for TB treatment are classified into two classes, first-line drugs and second-line drugs. First-line drugs are mainly bactericidal and combine a high degree of efficacy with a relatively low toxicity to the patient during treatment. These drugs include isoniazid, rifampicin, streptomycin, ethambutol, pyrazinamide, and fluoroquinolones. Second-line drugs are mainly bacteriostatic, which have a lower efficacy and are usually more toxic. These drugs include *para*-aminosalicylic acid, ethionamide, and cycloserine [2]. Drug-sensitive tuberculosis can be effectively treated with the combination of potent bactericidal agents including streptomycin or ethambutol, isoniazid, rifampin and pyrazinamide [2,3,4,5]. However, the successful treatment using drug combinations has been diminished by the outbreak of multidrug resistant tuberculosis (MDR-TB), defined as resistant to at least isoniazid and rifampicin. More recently, there is a new class of MDR, extensively drug-resistant TB (XDR-TB) defined as resistant to isoniazid and rifampicin and at least three of the six main classes of second-line drugs [6]. Furthermore, a more complex treatment of TB is associated with co-infection between M. tuberculosis and HIV (TB/HIV) [7,8,9,10]. Accordingly, the design of new and more potent antitubercular drugs for the management of drug-sensitive and drug-resistant TB is imperative. 

Isoniazid (INH) has the greatest bactericidal activity and is used almost from the outset of tuberculosis chemotherapy [11,12,13]. This antibiotic inhibits a 2-*trans*-enoyl-acyl carrier protein reductase (InhA) displaying a long-chain fatty acid elongation activity. Inhibition of this activity by INH blocks the biosynthesis of mycolic acids, which are major lipids of the mycobacterial envelope [14,15,16,17]. INH is a prodrug requiring the activation function of catalase-peroxidase (KatG) to generate the active form [18,19,20,21,22]. The reactive species generated from the activation process forms a covalent adduct with NAD+ that is a potent inhibitor of InhA [20,23,24]. INH-NAD adduct is a slow tight-binding competitive inhibitor of InhA that binds with an overall dissociation constant of 0.75 nM [25]. To reveal a better understanding of the isoniazid drug mechanism, the crystal structures of InhA in complex with NADH cofactor and INH-NAD adduct were isolated [15,26,27,28]. Although INH-NAD is an extremely potent InhA inhibitor, its ability for inhibiting InhA is diminished by drug resistance [29,30,31]. Therefore, the basic research in molecular biology for developing new and more effective InhA inhibitors is desirable. Recently, molecular docking and molecular dynamics simulations have been performed to study the binding of isoniazid onto the active site of InhA in an attempt to address the mycobacterial resistance against the drug [32]. Ligand-based drug design approaches have also been successfully employed to identify crucial features of INH derivative prodrugs as InhA inhibitors [33,34,35,36]. Based on the chemical structures of INH derivative prodrugs, they probably bind with NAD^+^ to form covalent adducts like the parent INH. Therefore, insight into the nature of these bioactive forms of INH derivative prodrugs in the InhA binding pocket should provide more detailed information for designing agents that greatly inhibit InhA. In the present study, molecular docking and quantum chemical calculations were employed to elucidate the potential antitubercular binding modes and main interactions of INH derivative adducts. In order to understand the structural requirement of InhA inhibitors, the relationship between structure and activity of these compounds was elucidated by CoMFA and CoMSIA methods [37,38]. The integrated results should aid in the rational design of InhA inhibitors with high potential antitubercular activity.

## 2. Results and Discussion

### 2.1. Validation of the molecular docking calculations

**Figure 1:**
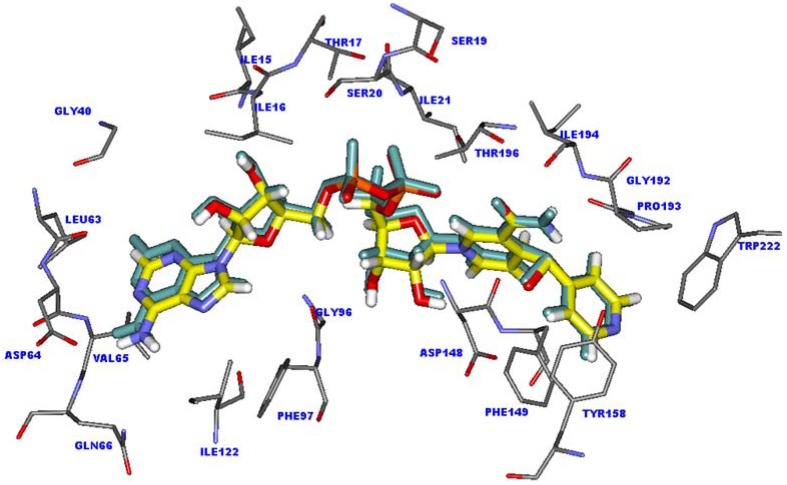
Superimposition of the X-ray crystal structure (carbon atoms colored by yellow) and docked conformation (green) of the INH-NAD adduct in the InhA binding pocket.

Molecular docking calculations were employed to predict the potential binding mode of INH derivative adducts in the InhA binding pocket. To ensure that the binding modes of adduct inhibitors obtained from molecular docking calculations are reliable the docking parameters in Autodock 3.05 program were validated. The structure of the INH-NAD adduct in the X-ray crystal structure was extracted and docked back into the binding pocket. The superimposition between the docked conformation and the X-ray crystal structure of INH-NAD adduct is shown in Figure 1. The docked conformation of INH-NAD adduct is close to the binding mode found in the X-ray crystal structure with rmsd of 0.44 Å, indicating that the docking parameters are reasonable to generate the binding mode of INH-NAD adduct in the InhA binding pocket. Therefore, molecular docking calculations could be extended to search the binding modes of INH derivative adducts in the data set.

### 2.2. Molecular docking analysis of the highly active compounds

INH and its derivatives were used for molecular docking calculations. The chemical structures and experimental biological activities against the BCG strain of *M. tuberculosis* of these compounds are shown in Table 1. Among the data set, INH shows the highest activity against strains of *M. tuberculosis* with a log (1/MIC) value of 7.70, where MIC is the *in vitro* minimum inhibitory concentration in µg/mL unit. The docked conformation of the INH-NAD adduct into the active site of InhA is indeed similar to that observed in the X-ray crystal structure. The 4-pyridyl ring of the INH-NAD adduct is buried in the cavity formed by the Ala191, Gly192, Trp222, Tyr158, Phe149 and Pro193 residues, as shown in Figure 1. This ring forms a π-π interaction with the aromatic sidechain of Phe149 and a H-π interaction with the aromatic sidechain of Tyr158. For the nicotinamide part, two hydrogen bonds with the carbonyl oxygen backbone of Asp148 and van der Waals interactions with Ile21, Ala191, Gly192, Pro193, Thr196 and Met199 could be observed. The pyrophosphate part is held strongly by the hydrogen bonding interaction of surrounding amino acids in the InhA binding site. Two oxygen atoms of the pyrophosphate form two hydrogen bonds with the OH group of Thr196. Moreover, two hydrogen bonds between the NH backbone of Ile21 and the OH sidechain of Ser20 with phosphate oxygens are also present. A hydrogen atom of NH_2_ and a nitrogen atom of the adenine part interact with the carbonyl oxygen group of Asp64 and the NH backbone of Val65 to form two hydrogen bonds. According to the observed interactions of INH-NAD adduct, the main interactions found in the binding are hydrogen bond interactions, therefore they may play an important role in the binding of adduct inhibitors.

**Table 1:** The chemical structures and experimental biological activities against the BCG strain of *M. tuberculosis* of INH derivatives taken from literature [39].

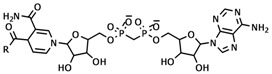					
Compound	R	Log (1/MIC)	Compound	R	Log (1/MIC)
INH	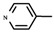	7.70	18	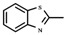	5.10
1	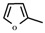	7.22	19 ^[a]^	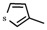	5.10
2^[a]^	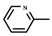	6.82	20	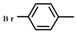	4.92
3	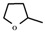	6.52	21^[a]^	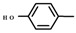	4.92
4	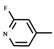	6.40	22	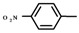	4.92
5	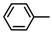	6.22	23	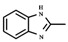	4.82
6	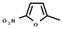	6.10	24	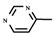	4.70
7	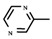	5.82	25	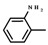	4.52
8	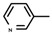	5.70	26^[a]^	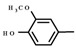	4.52
9^[a]^	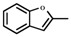	5.70	27	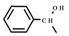	4.40
10^[a]^	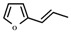	5.52	28	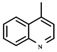	4.10
11	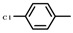	5.52	29	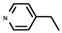	4.00
12	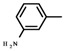	5.52	30	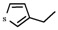	4.00
13	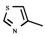	5.52	31	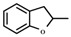	3.65
14	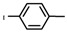	5.22	32	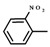	3.52
15	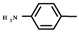	5.22	33	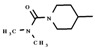	3.22
16	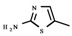	5.22	34	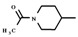	3.22
17	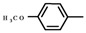	5.10	35	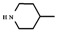	3.22

^[a]^ The test set compounds for CoMFA and CoMSIA.

**Figure 2:**
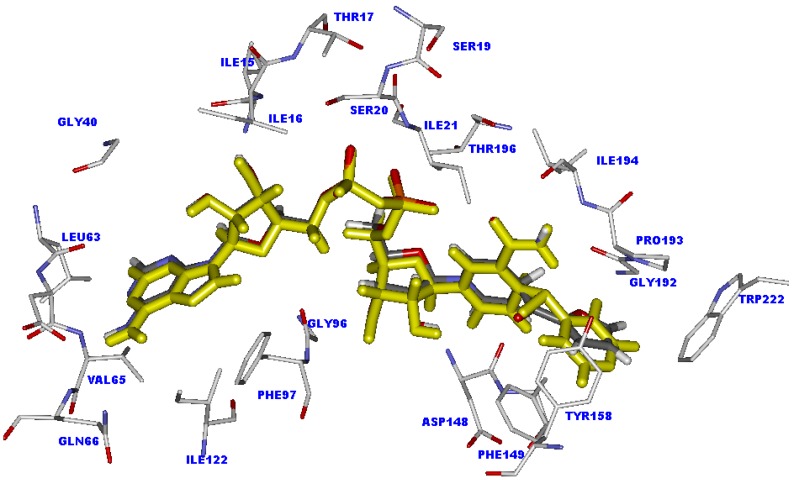
Superimposition of the INH-NAD adduct (colored by yellow) and compound **1** (colored by atom type) in InhA binding pocket obtained from molecular docking calculations.

With regard to compound **1** possessing a furan ring at the R substituent position, the inhibitory activity differs slightly from the activity of INH, with log (1/MIC) of 7.22. The NAD part of compound **1** is aligned well with the NAD part of INH, as shown in Figure 2. The hydrogen bond distances of the NAD part of compound **1** are insignificantly changed (< 0.1 Å), compared with INH, as shown in Table 2. Because of the smaller R substituent of compound **1** as compared with the 4-pyridyl ring of the INH adduct, the π-π interaction with the aromatic sidechain of Phe149 is lost. However, a H-π interaction with the aromatic sidechain of Phe149 is formed to compensate for the lost interaction. These results reinforce the slight difference on the inhibitory activity of compound **1** as compared with INH.

**Table 2:** The hydrogen bond distances (Å) between the NAD part of all adducts and amino acid residues.

Cpd.	Nicotinamide	Pyrophosphate			Adenine ring	
	Asp148	Thr196	Ser20	Ile21	Asp64	Val65
INH	2.75, 2.34	2.99, 3.66	2.47	2.69	2.09	2.80
1	2.74, 2.33	2.92, 3.65	2.42	2.63	2.14	2.82
2	2.91, 2.40	2.97, 3.66	2.43	2.72	2.12	2.74
3	2.80, 2.36	2.85, 3.59	2.42	2.59	2.21	2.86
4	2.87, 2.36	2.94, 3.64	2.43	2.68	2.16	2.76
5	2.91, 2.41	2.89, 3.62	2.41	2.66	2.25	2.76
6	2.79, 2.35	2.91, 3.65	2.40	2.62	2.13	2.81
7	2.87, 2.34	2.91, 3.68	2.41	2.64	2.23	2.75
8	2.95, 2.39	2.90, 3.61	2.43	2.67	2.25	2.76
9	3.71, 3.38	3.21, 4.53	2.13	3.22	1.64	2.45
10	2.93, 2.99	3.31, 4.38	2.28	3.14	1.56	2.43
11	3.29, 2.81	3.10, 3.77	2.30	2.90	1.78	2.65
12	3.01, 2.50	2.91, 3.68	2.41	2.73	2.16	2.71
13	2.87, 2.34	2.90, 3.67	2.41	2.63	2.22	2.76
14	3.46, 3.15	3.12, 3.88	2.14	2.97	1.67	2.60
15	3.20, 2.85	2.87, 3.74	2.29	2.82	1.88	2.55
16	3.09, 2.53	3.12, 3.84	2.39	2.86	1.89	2.58
17	2.67, 2.99	3.31, 4.53	2.28	3.14	1.56	2.43
18	3.83, 3.62	3.30, 4.71	2.11	3.34	1.72	2.59
19	2.82, 2.36	3.02, 3.64	2.83	2.73	2.35	2.86
20	3.38, 2.89	3.08, 3.83	2.21	2.89	1.77	2.59
21	3.28, 2.56	3.10, 3.67	2.30	2.90	1.78	2.65
22	3.38, 3.03	2.82, 3.76	2.18	2.82	1.81	2.58
23	3.55, 3.51	3.43, 4.66	2.19	3.37	1.53	2.60
24	2.85, 2.33	2.91, 3.67	2.41	2.64	2.23	2.75
25	2.72, 2.39	2.89, 3.70	2.41	2.66	2.25	2.76
26	3.25, 2.81	2.90, 3.70	2.38	2.88	1.92	2.55
27	2.78, 2.95	2.89, 3.66	3.01	3.15	2.15	2.61
28	3.06, 2.77	3.42, 5.17	2.15	3.07	2.62	2.26
29	2.35, 2.24	3.02, 3.66	2.91	2.70	2.50	2.93
30	2.34, 2.23	3.02, 3.56	2.83	2.73	2.35	2.86
31	4.66, 3.79	3.17, 4.46	2.32	3.35	2.15	2.69
32	2.25, 2.38	3.05, 3.68	2.70	2.71	2.09	2.78
33	3.90, 3.07	2.73, 3.57	2.16	2.81	2.02	2.68
34	3.52, 3.16	3.45, 5.03	2.19	3.19	1.96	2.59
35	2.93, 2.42	2.99, 3.68	2.42	2.74	2.01	2.75

**Figure 3:**
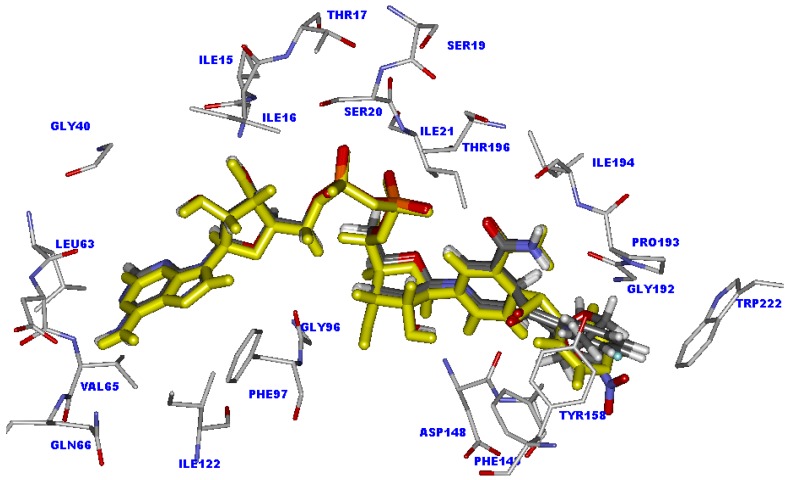
Superimposition of the INH-NAD adduct (colored by yellow) and compounds **2-7** (colored by atom type) in InhA binding pocket obtained from molecular docking calculations.

For the other highly active compounds, compounds **2**-**7**, the NAD parts of these compounds lie in the same position as the NAD part of INH, as shown in Figure 3. The important hydrogen bonds of these compounds are maintained, but the preferable interactions of the R substituents of Ala191, Gly192, Trp222, Tyr158, Phe149 and Pro193 in the cavity are eliminated.

### 2.3. Docking analysis of the moderately active compounds

The different R substituents of the INH derivative adducts are located inside the hydrophobic cavity generated by the residues of Ala191, Gly192, Trp222, Tyr158, Phe149 and Pro193. The R substituents of moderately active compounds, compounds **8-23**, mostly interact with Pro193 to form hydrophobic interactions and interact with Ala191, Gly192 and Tyr158 to form van der Waals interactions. Unfavorably, a steric effect between the R substituents and the aromatic sidechain of Trp222 could be observed. Two of six member ring substituents, phenyl and pyridyl rings, are favored to form a hydrophobic interaction with Pro193, such as compounds **12**, **15**, **17** and **20**. However, the bulkier atoms or group of atoms in the six member ring substituents could collide with surrounding amino acid, in particular with Trp222. To reduce the steric effect, the position of the NAD part of these compounds may be shifted as compared with INH, leading to an increase in the hydrogen bond distances of the NAD part, especially for the two hydrogen bonds of the nicotinamide ring with Asp148, as shown in Table 2.

For the five member ring substituents contained in compounds **13** and **19**, no steric effect could be found with the surrounding amino acids in the R substituent cavity. The important hydrogen bonds of the NAD part also remain in the same quality as in INH, as shown in Table 2. Because the five member ring substituents of these compounds are rather small, the favorable interactions for high inhibitory activity, H-π or π-π interactions with Phe149 and H-π interaction with Tyr158, could not be formed. As for the interaction analysis of the moderately active compounds in the InhA binding pocket, the loss of inhibitory activity of these compounds could be explained by the steric effect and the absence of some favorable interactions in the R substituent cavity.

### 2.4. Docking analysis of the weakly active compounds

Compounds **24**-**35** show lower activity against strains of *M. tuberculosis*. The van der Waals interactions are the major interaction of the R substituents of these compounds. Steric effects between the substituents and the sidechain of Pro193, Phe149, Tyr158 and Trp222 could be found. The bicyclic substituents in compounds **28**, **31** seem to be the large substituents. These large substituents bump into amino acids in the R substituent cavity, leading to a shift in the position of the NAD part of these compounds, compared with INH. Moving the NAD part position leads to a loss of the hydrogen bond interactions of the nicotinamide ring with Asp148 and pyrophosphate part with Thr196, as shown in Table 2.

Compounds **33**-**35** display the lowest activity against strains of *M. tuberculosis*. The R positions of these compounds are substituted by a piperidine ring. The bulky piperidine ring could only form a van der Waals interaction with amino acids in the R substituent cavity. A steric effect of this substituent with the sidechains of Pro193, Phe149, Tyr158 and Trp222 is also observed. The bulky groups at the N position on piperidine ring of compounds **33**-**35** reinforce the steric effect of these compounds. Because of the steric effect, the hydrogen bonding interactions of nicotinamide part with Asp148 and pyrophosphate part with Thr196 of compounds 33, 34 are eliminated, as shown in Table 2. 

**Figure 4:**
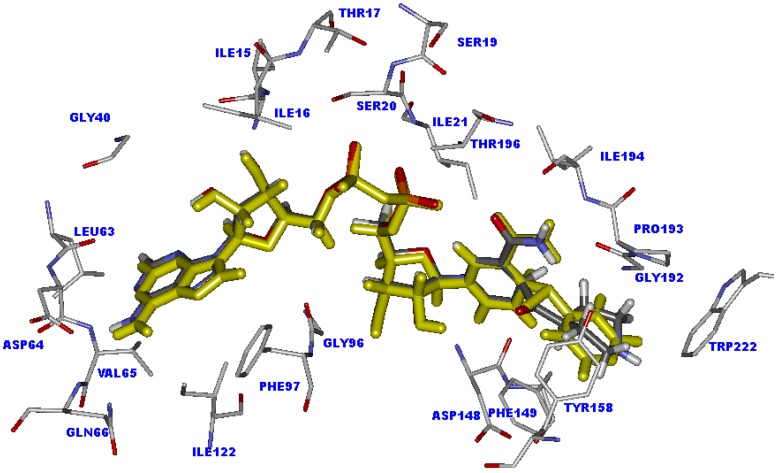
Superimposition of the INH-NAD adduct (colored by yellow) and compound **35** (colored by atom type) in the InhA binding pocket obtained from molecular docking calculations.

Interestingly, although compound **35** possesses the piperidine ring in the R substituent, the NAD part of this compound shows good alignment with the NAD part of the INH-NAD adduct, as shown in Figure 4. The piperidine ring interacts with Phe149, Tyr158 and Trp222 to form H-π interactions. Moreover, the steric effect is absent in this compound. The important hydrogen bonding interactions of the NAD part are well within the range of INH, as presented in Table 2. Therefore, the weak inhibitory activity of compound **35** may be related to the activation process. The activation process of these compounds proceeds via free radical formation. The ability of R substituents to stabilize the acyl radical should influence the efficiency for coupling of the acyl radical with NAD+ to form an adduct inhibitor for InhA. The rich aromatic group has a higher capacity to stabilize the acyl radical than a non-aromatic group. The electronic charge of the acyl radical can be delocalized through resonance of the aromatic ring. This makes it more stable, whereas the electronic charge delocalization of acyl radical in the non-aromatic system could not occur. Therefore, the aromatic pyridyl ring of INH shows higher potency to stabilize the acyl radical than the non-aromatic piperidine ring of compound **35**, thereby explaining the lower inhibitory activity of this compound, compared with INH. Accordingly, the low potency for stabilizing the acyl radical of R substituents may be additional cause for the poor activity against *M. tuberculosis* of other compounds that contain the R substituent like compound **35**.

### 2.5. The favorable interactions for binding of INH derivative adducts

The hydrogen bonds of the NAD part with Asp148, Thr196, Ile21, Ser20 and Asp48 are major interactions for adduct binding. For the R substituent, the π-π or H-π interaction with the aromatic sidechain of Phe149 and the H-π interaction with the aromatic sidechain of Tyr158 are also required to enhance the binding of these inhibitors. Moreover, the capacity of R substituents for stabilizing the free radical intermediate in the activation process is interesting. To facilitate the activation process, R substituents should reveal a high aromatic property. Importantly, R substituents must not present a steric effect in the R substituent cavity. The 4-pyridyl ring and furan ring seem to be optimal for binding of InhA inhibitors. The replacement of hydrogen atoms on these ring with bulkier atoms or groups of atoms such as Cl, I, Br, NH_2_ and NO_2_ completely reduces the inhibitory activity of compounds because of their steric effect in the R substituent cavity.

### 2.6. Interaction energy of the INH-NAD adduct in the InhA binding pocket

To investigate the key interactions for binding of INH-NAD adduct and its derivatives, INH, the highly active compound **1** and the weakly active compound **33** were selected for quantum chemical calculations. Moreover, to explain the effect of the activation process on the inhibitory activity of these inhibitors, the interaction energy of compound **35** was also calculated. Based on quantum chemical calculations at MP2/6-31G(d) level of theory, the interaction energies of INH-NAD adduct fall in the range of -33.01 to 13.20 kcal/mol. The 4-pyridyl ring, the R substituent of INH-NAD adduct, shows an attractive interaction with Phe149 and Tyr158 with an attraction energy of 1.21 and 2.09 kcal/mol. These attraction energies correlate well with the observed interactions from the X-ray crystal structure of INH-NAD bound in the InhA active site. The 4-pyridyl ring forms a π-π interaction with the aromatic sidechain of Phe149 and a H-π interaction with the aromatic sidechain of Tyr158. The nicotinamide part provides the main attraction energy of 4.31 kcal/mol with Asp148. This attraction energy agrees with the weak hydrogen bonds produced from two hydrogen atoms on the nicotinamide ring and the carbonyl backbone of Asp148. In this part, a high repulsion interaction with Ile194 could be observed with a repulsion energy of 13.20 kcal/mol. From the X-ray crystal structure, the carbonyl amide of the nicotinamide part lies near the carbonyl backbone of Ile194. The repulsion between two groups with the same charge may occur. Nicotinamide ribose displays a highly attractive interaction of 11.15 kcal/mol with Lys165, due to the electrostatic interaction of nicotinamide ribose hydroxy group with Lys165. The pyrophosphate part presents higher attraction energy than other parts of the INH-NAD adduct. The highest attraction energy of 33.01 kcal/mol is found between the pyrophosphate part and Ser20. Moreover, other highly attractive interactions are also produced between the pyrophosphate part and Ile21 and Thr196, with attraction energies of 23.92 and 24.48 kcal/mol, respectively. These high attraction energies of the pyrophosphate part relate to the strong hydrogen bond interactions of the phosphate oxygens with Ser20, Thr196 and Ile21. These results confirm that the hydrogen bond interactions are the key interactions in this region. Among all attraction energies of the adenine part, the interaction with Asp64 shows the highest attraction energy (9.11 kcal/mol), consistent with the hydrogen bond interaction of NH_2 _on the adenine part with the carbonyl sidechain of Asp64. Indeed in the X-ray crystal structure, the NH backbone of Val65 can form a hydrogen bond with the nitrogen atom of the adenine ring. The interaction energy of this interaction is low (2.67 kcal/mol). Based on the interaction energy calculation results, all high attraction energies correlate with the hydrogen bond interactions of the INH-NAD adduct in the InhA binding pocket observed in the X-ray crystal structure. Therefore, we can conclude that the hydrogen bond interactions are crucial for binding of the INH-NAD adduct, particularly the hydrogen bond interactions of the pyrophosphate part.

### 2.7. Interaction energy of the highly active compounds in the InhA binding pocket

In the data set of our study, the INH-NAD adduct is the highest potency compound against *M. tuberculosis*, with a log (1/MIC) of 7.70. The inhibitory activity of compound **1** at a log (1/MIC) of 7.22 is comparable with INH-NAD adduct. These two compounds show structural difference in the R substituent. The furan and 4-pyridyl rings are maintained at the R substituent position of compound **1** and the INH-NAD adduct, respectively. The binding mode of compound **1** in the InhA active site was studied by means of molecular docking calculations. To quantitatively elucidate the R substituent influence on its activity, the interaction energy of compound **1** was calculated and compared with the calculated interaction energy of the INH-NAD adduct. The interaction energy of compound **1** is presented in Table 3. The furan ring of compound **1** attractively contacts with Phe149 with an attraction energy of 0.93 kcal/mol. This attractive interaction correlates with the H-π interaction of the hydrogen atom on the furan ring with the aromatic sidechain of Phe149. Moreover, this attractive interaction is comparable with the π-π interaction of the 4-pyridyl ring of INH-NAD adduct with the aromatic sidechain of Phe149. Additionally, slightly attractive interactions with Pro193 and Trp222 could be found with attraction energies of 0.89 and 1.48 kcal/mol, respectively. However, the furan ring of compound **1** is missing the attractive interaction with Try158 of about 1 kcal/mol. This result correlates with the docking results in which the smaller furan ring loses the H-π interaction with Try158 compared with the 4-pyridyl ring of the INH-NAD adduct. For other parts of compound **1**, nicotinamide, nicotinamide ribose, pyrophosphate, adenine ribose and adenine ring, the main interaction energies are all similar to those of the INH-NAD adduct. These results suggest that the slightly lower inhibitory activity of compound **1** as compared with INH-NAD adduct could be attributed to the loss of attractive interaction of the furan substituent with Try158 because of its smaller size.

**Table 3:** Interaction energy between each part of the adducts of INH, compounds 1, 33, 35 and each amino acid surrounding within 6 Å from each part of adducts in InhA binding pocket.

Adduct fragment	Amino acid	Interaction energy (kcal/mol)			
		INH	Cpd. 1	Cpd. 33	Cpd. 35
R substituent	Phe149	-1.21	-0.93	30.17	2.64
	Tyr158	-2.09	-0.98	23.02	-0.07
	Ala191	0.78	3.69	18.01	4.01
	Gly192	1.61	1.96	-2.92	5.13
	Pro193	0.23	-0.89	23.68	6.72
	Trp222	0.03	-1.48	18.17	-0.21
Nicotinamide	Ile21	-0.62	0.54	-0.61	-0.39
	Met147	-0.58	-0.23	-0.08	-0.32
	Asp148	-4.31	-5.98	-4.65	-5.75
	Phe149	6.95	2.50	-0.20	4.55
	Lys165	1.56	1.90	3.43	2.64
	Ala191	-2.41	-1.84	-1.10	-1.90
	Gly192	-0.12	-0.57	-0.24	-0.35
	Pro193	0.52	0.62	-0.71	-0.22
	Ile194	13.20	10.70	3.34	3.74
	Thr196	-1.88	-1.88	-2.66	-2.09
Nicotinamide Ribose	Gly14	-0.18	-0.21	-0.21	-0.20
	Ser20	0.01	0.01	-0.02	0.01
	Ile21	-0.54	0.29	0.39	-0.19
	Ala22	-0.02	-0.04	-0.04	-0.03
	Ser94	-1.30	-0.41	-1.71	-1.15
	Ile95	0.22	-0.32	0.00	-0.17
	Gly96	-1.19	-1.08	-1.21	-1.24
	Met147	0.54	3.76	-1.27	0.99
	Asp148	-0.29	-0.61	0.26	-0.39
	Phe149	-0.26	-0.23	-0.22	-0.24
	Met161	-0.18	-0.16	-0.18	-0.16
	Lys165	-11.15	-11.87	-10.19	-12.00
	Ala191	-0.30	-0.31	-0.27	-0.30
Pyrophosphate	Gly14	8.87	9.29	9.79	9.34
	Ile16	-10.17	-10.28	-5.55	-10.12
	Thr17	-7.32	-7.81	-9.80	-7.83
	Ser19	-1.09	-1.24	-1.77	-1.24
	Ser20	-33.01	-32.55	-13.41	-32.08
	Ile21	-23.92	-22.51	-23.39	-23.87
	Ala22	-10.86	-11.12	-10.26	-10.84
	Ser94	6.63	9.15	7.41	8.70
	Ile95	-4.14	-4.10	-4.19	-4.16
	Gly96	3.27	3.35	3.41	3.41
	Met147	-3.63	-3.57	-2.99	-3.49
	Thr196	-24.48	-24.93	-25.04	-24.94
Adenine Ribose	Gly14	2.62	6.28	6.74	6.42
	Ile15	-1.36	-1.32	-1.32	-1.37
	Ile16	3.17	3.92	2.92	2.96
	Ser20	-0.09	-0.03	-0.02	-0.04
	Phe41	0.10	0.27	3.00	0.65
	Val65	0.04	0.06	0.07	0.06
	Ser94	-0.15	0.17	-0.10	0.07
	Ile95	-2.08	-1.55	-1.88	-1.64
	Gly96	-1.74	-1.79	-1.36	-1.70
	Phe97	-0.50	-0.36	-0.35	-0.36
	Ile122	0.11	0.12	0.11	0.12
Adenine	Gly14	-0.65	-0.80	-0.71	-0.76
	Gly40	-1.36	-0.20	-0.13	-0.18
	Phe41	-2.03	-2.48	-2.35	-2.45
	Leu63	0.18	1.32	1.51	1.67
	Asp64	-9.11	-8.43	-7.54	-8.80
	Val65	-2.67	4.18	14.11	5.76
	Gln66	0.85	1.31	1.66	1.45
	Ile95	-0.95	0.45	0.90	0.15
	Gly96	-1.31	-1.18	-0.88	-1.08
	Phe97	-0.03	0.05	0.04	0.05
	Ile122	-0.94	-0.30	-0.93	-0.70

### 2.8. Interaction energy of the weakly active compounds in the InhA binding pocket

The docked structure of compound **33**, the least active compound, was taken to calculate the interaction energies and then these calculated interaction energies were compared with the interaction energy of the INH-NAD adduct, the most active compound. The interaction energy of this compound is given in Table 3. Compound **33** possesses a piperidyl ring in the R substituent position. The interactions of the piperidyl ring substituent with Phe149, Tyr158, Ala191, Pro193 and Trp222 show high repulsion energies (18.01-23.68 kcal/mol). These high repulsion energies relate to the steric effect of the R substituent, as found from the docking results. As in the docking results, the steric effect changes the position of the NAD part of compound **33** leading to an alteration of the hydrogen bond patterns of this compound. Supporting the molecular docking results, the attraction energy of the important hydrogen bonds between pyrophosphate and Ser20 are decreased about 20 kcal/mol as compared with the INH-NAD adduct. 

Moreover, the attraction energy of pyrophosphate with Ile16 is also reduced by about 5 kcal/mol. In addition of the reduction of the main attraction energy, a repulsion energy of 14.11 kcal/mol between the adenine ring and Val65 is additionally detected. Therefore, based on the calculated interaction energy, the high repulsion energy between the R substituent and surrounding amino acid could account for the lowest activity of compound **33**. 

Compound **35** shows inhibitory activity like compound **33** with log (1/MIC) of 3.22. The binding mode of this compound is similar to that of the INH-NAD adduct, as described in the previous discussion of the docking results. To elucidate the important binding interactions of this compound, the interaction energy of compound **35** was calculated as given in Table 3. The piperidyl R substituent of compound **35** exhibits slightly attractive interactions with Tyr158 and Trp222. Repulsive interactions of the piperidyl ring with Phe149, Ala191, Gly192 and Pro193 could be observed with repulsion energy of less than 6.72 kcal/mol. Nevertheless, all interaction energies of other parts of compound **35** are quite comparable with those of INH-NAD. Moreover, the repulsion energy between the nicotinamide and Ile194 is decreased about 10 kcal/mol. Based on the calculated interaction energy, the binding affinities of compound **35** and INH in the InhA binding pocket should be similar. Therefore, the lower activity of compound **35** may depend on the activation process that requires the high capacity of substituents for stabilizing the free radical intermediate. In this case, the aromatic pyridyl rings of INH-NAD are more suitable for the activation process than the non-aromatic piperidyl ring of compound **35**, supporting the lower inhibitory activity of this compound.

### 2.9. CoMFA and CoMSIA models

The statistical parameters of the best CoMFA and CoMSIA models generated based on molecular docking alignment are shown in Table 4. The obtained CoMFA and CoMSIA models are consistent with good correlation and predictive capability, the non-cross-validation (r^2^) and the cross-validation (r^2^_cv_) of 0.94 and 0.67 for CoMFA model and 0.96 and 0.74 for CoMSIA model. The selection of the best CoMSIA is based on the highest r^2^_cv_. Thus, the best CoMSIA model includes steric and electrostatic fields. Considering all statistical parameters, the best model CoMSIA including steric and electrostatic fields shows slightly more predictive ability than the best CoMFA model. 

**Table 4:** The statistical parameters of the CoMFA and CoMSIA models.

Models	Statistical parameters						Fraction
	r^2^_cv_	r^2^	N	s-press	SEE	F	
CoMFA	0.67	0.94	5	0.71	0.31	72.74	91/9 (S/E)
CoMSIA							
**S/E**	**0.74**	**0.96**	**6**	**0.64**	**0.25**	**82.78**	**43/57**
S/E/H	0.55	0.87	5	0.86	0.46	28.30	17.4/21.2/61.4
S/E/HD	0.56	0.93	6	0.86	0.35	42.94	27.1/28.6/44.3
S/E/HA	0.62	0.93	6	0.77	0.34	41.26	32.2/38.9/28.9
S/E/HD/HA	0.38	0.95	5	1.14	0.28	86.84	18.2/22.9/39.3/19.6
/E/H/HD/HA	0.32	0.91	5	1.06	0.40	39.84	11.1/13.4/40.5/26.3/8.7

Bold values indicate the best CoMSIA model. r^2^_cv_, leave-one-out (LOO) cross-validated correlation coefficient; r^2^, non-cross-validated correlation coefficient; N, optimum number of components; s-press, Standard error of prediction, SEE, standard error of estimate; F, F-test value; S, steric field; E, electrostatic field; H, hydrophobic; HD, hydrogen donor field and HA, hydrogen acceptor field.

**Figure 5:**
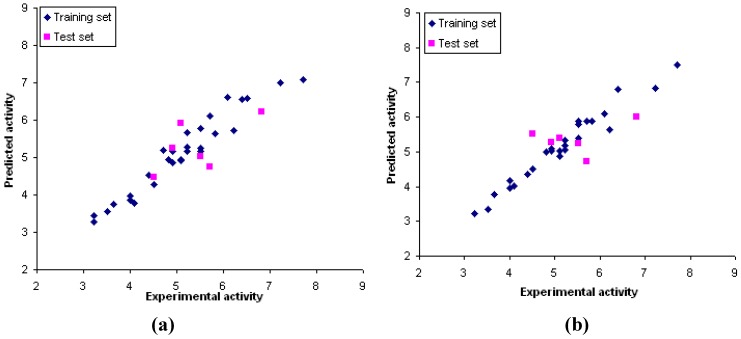
Plots between the experimental and predicted activities of training and test sets from CoMFA model **(a)** and CoMSIA model **(b)**.

The correlations between experimental and predicted activities are shown in Figure 5. These results exhibit a good agreement between the experimental and predicted values. In order to verify the predictive ability of the obtained models, the biological activities of the test set were predicted by these CoMFA and CoMSIA models. All compounds show predicted values within one logarithmic unit difference from the experimental values. These results show that CoMFA and CoMSIA models provide good accuracy for predicting the inhibitory activity.

### 2.10. CoMFA and CoMSIA contour analysis of INH derivative adducts

**Figure 6:**
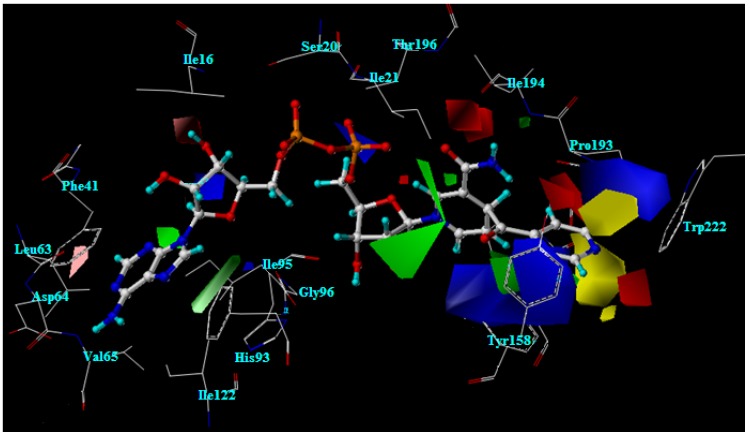
CoMFA contour maps for steric and electrostatic fields.

**Figure 7:**
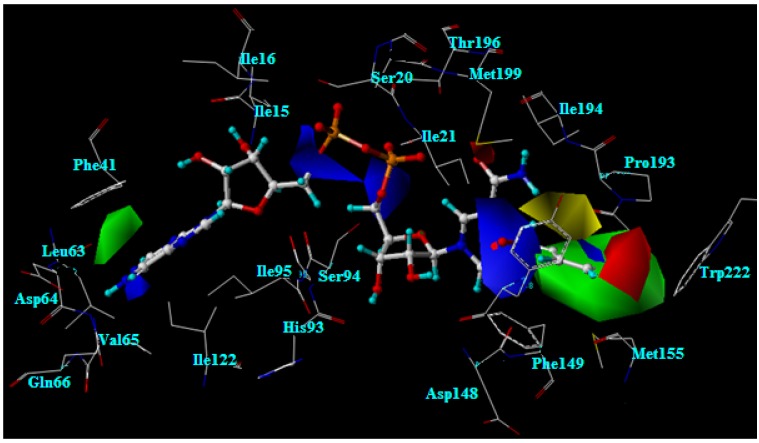
The best CoMSIA contour maps for steric and electrostatic fields.

The CoMFA and CoMSIA models reveal the importance of steric and electrostatic fields through the contour maps shown in Figure 6 and Figure 7. Favorable and unfavorable steric interactions are displayed in green and yellow contours, respectively, while blue and red contours illustrate the regions that favor the positive and negative charge, respectively. The CoMSIA steric and electrostatic contours appear more localized and detailed than those of the CoMFA model because they are closer to ligand. 

### 2.11. The steric contour analysis of INH derivative adducts

The CoMFA steric field reveals two yellow contours and one large green contour near the R substituent, as shown in Figure 6. Meanwhile, the CoMSIA steric field reveals one yellow contour above the plane of the R substituent. The R substituent is buried in the large green contour, as presented in Figure 7. However, steric contours from two models agree that a large plane of the R substituent is preferable in producing higher activity against strains of *M. tuberculosis*. Nevertheless, the R substituent size is limited by the neighboring steric region. As the X-ray structure of the INH-NAD adduct bound in InhA, three amino acid residues, Ala191, Gly192 and Trp222 are located near the steric region. Therefore, any larger R substituent may collide with these residues. From molecular docking calculations, the moderately active compounds and weakly active compounds display steric effects with Trp222.

Compounds **1**, **3**, **6**, **13** and **16** that contain a five member ring substituent show lower activity than INH bearing the 4-pyridyl ring. The five member ring of compound **1** is completely buried in the large green contour but is not present any atom in the steric region represented by a yellow contour. As compared with the 4-pyridyl plane of INH, the plane of the five member ring is rather small. The results suggest that the plane size of the five member ring substituent does not generate any steric effect and it is not large enough for improving the inhibitory activity. Compounds **9**, **18**, **23**, **25**, **28**, **31** and **32** possess the large plane of bicyclic rings at the R substituent position. Although the bicyclic rings of these compounds are favorable for a green contour, the activities of these compounds are not better as compared with INH. From molecular docking calculations, these substituents are too large, which leads to generation of a steric effect with Trp222. As a result of the steric hindrance, the hydrogen bond interactions of the pyrophosphate parts of these compounds with Thr196 are eliminated. Compounds bearing the piperidine ring, **33-35**, show the lowest activity. The piperidine ring of these compounds is bulkier than the 4-pyridyl ring of INH. The piperidine ring of compound 34 lies in the yellow contour, resulting in unfavorable active compound against strains of *M. tuberculosis*. These results indicate that steric effect is an additional cause of the lower activity of these compounds. 

### 2.12. The electrostatic contour analysis of INH derivative adducts

The CoMFA contours of the electrostatic field are shown in Figure 6. Blue and red contours are located near the R substituent, the 4-pyridyl ring of the INH-NAD adduct. Two large blue contours are present outside the plane of the 4-pyridyl ring. Red contours lie above the 4-pyridyl plane and another red contour lies opposite the nitrogen atom at the 4-pyridyl ring of the INH-NAD adduct (N4). In the case of the CoMSIA contour, the electrostatic field contours are closer to the ligand than in the CoMFA contour, as shown in 7. A large red contour covers N4 in the 4-pyridyl ring of the INH-NAD adduct. Two blue contours are present outside the plane of the 4-pyridyl ring. Therefore, to improve the activity of adduct inhibitors based on CoMFA and CoMSIA models, the N4 position should contain the electronegative group and both sides of the R substituent plane should contain electropositive groups. Hydrogen atoms at the 4-pyridyl ring of INH-NAD adduct lie near both blue contours as shown in Figure 7. One of these hydrogen atoms is replaced by a fluorine atom in compound **4**. The results show that the fluorine atom does not enhance the inhibitory activity of this compound because this atom is close to the unfavorable blue contour. In accordance with the CoMFA and CoMSIA models for improving the inhibitory activity, hydrogen atoms on both sides of the 4-pyridyl ring should be substituted by more electropositive groups. The phenyl ring of compound **5** is like the 4-pyridyl ring of INH except for the fact the nitrogen atom (N4) of the 4-pyridyl ring is replaced by the carbon atom of a phenyl ring (C4). The alignment of compound **5** and the INH-NAD adduct obtained from molecular docking calculations reveals that the N4 of the pyridyl ring lies in the same position as C4 of the phenyl ring. The hydrogen atom attached to the C4 of the phenyl ring of compound **5** is placed in the red contour. Regarding inhibitory activity, compound **5** shows lower inhibitory activity than INH. These results imply that the hydrogen atom attached to the C4 of the phenyl ring is not favourable. Compounds **11**, **14**, **20** and **22** contain R substituents like compound **5**. The C4 of the phenyl ring of these compounds attaches to Cl, I, Br and NO_2_ that are more electronegative than the hydrogen atom in compound **5**. Although these groups are favorable for a red contour, the activities of these compounds are not increased as compared with compound 5. From molecular docking calculations, these compounds lose the important hydrogen bonds of the pyrophosphate moiety because of the steric effect of Cl, I, Br and NO_2_ with Trp222. These results imply that the nitrogen atom on the 4-pyridyl ring is optimal for both the steric and electrostatic fields.

### 2.13. The structural requirement of the R substituent of INH derivative adducts

To enhance the activity of INH derivative adducts, the R substituents should contain a large plane. Both sides of the plane should present electropositive groups. However, size of the R substituents is controlled by the steric region above the substituent plane. Accordingly, the steric and electrostatic fields of the 4-pyridyl ring are optimal for greater potency of compounds. The improvement of electrostatic properties on the 4-pyridyl ring by replacement of the hydrogen atom on both sides of the 4-pyridyl ring with more electropositive groups should be done carefully because a steric effect can also be generated.

## 3. Experimental

### 3.1. Biological activity data

Isoniazid and its 35 derivatives, a total of 36 compounds, with the general formula RC(O)NHNH_2_ were chosen from the original reference [39]. The inhibitory activities of INH and its derivatives tested against the BCG strain of *M. tuberculosis* were reported in Table 1. Because the structure-activity relationship of these compounds was investigated in this study, the inhibition activities have been expressed in terms of log (1/MIC). Isoniazid derivatives are separated into two groups; 30 compounds served as the training set and six compounds served as the test set, sampling from structurally diverse molecules possessing various ranges of logarithm unit activities.

### 3.2. Geometry optimization

In the present study, the chemical structures of INH derivatives are presented in the form of INH derivative adducts, the active forms of these inhibitors. The X-ray crystal structure of the INH-NAD adduct in the InhA enzyme (pdb code 2ZID) was used as the initial geometry to build of the active adducts of the INH derivatives. All chemical structures of adducts were drawn with the Gaussview 3.07 program [40]. The NAD part of all adducts was fixed. Only the variable R substituents of adducts were fully optimized using HF/3-21G method.

### 3.3. Molecular docking calculations

The X-ray crystal structure of the INH-NAD adduct complexed to InhA (pdb code 2ZID) was used for molecular docking calculations. Docking calculations were carried out by the Autodock 3.05 program using the Lamarckian Genetic Algorithm (LGA) [41]. The INH-NAD adduct was extracted from the complex structure and all hydrogen atoms were added. Only polar hydrogen atoms and Kollman charges were added to the protein using the AutoDockTools. Solvation parameters were generated on the protein using the Addsol utility of Autodock. The grid maps representing the protein in the actual docking process were calculated with Autogrid. The dimensions of the grids were 60 × 60 × 60 points with a spacing of 0.375 Å between the grid points and the center close to the ligand. Docking parameters were used as default values, except for the number of docking runs which was set to 50. The INH-NAD adduct was docked back into the InhA binding pocket to validate the docking method, then all derivative adducts were docked. During the docking process, the InhA enzyme and the NAD part of all adducts were kept rigid. Only the variable R substituents were allowed to freely rotate. The resulting docked conformations were those found to have the lowest final docked energy and the greatest number of members in the cluster, with a root mean square deviation (RMSD) cluster tolerance of 0.5 Å.

### 3.4. Interaction energy calculations

To elucidate the important interactions of INH derivative adducts in the InhA binding pocket, individual interaction energies between adducts and the surrounding amino acids were computed. The X-ray structure of the INH-NAD adduct, docked conformations of the highly active adduct (compound **1**) and the lowest active adducts (compounds **33** and **35**) were selected to calculate their interaction energies. The whole structure of the INH-NAD adduct and its derivatives was fragmented into small parts consisting of the R substituent, nicotinamide, nicotinamide ribose, pyrophosphate, adenine and adenine ribose. The ends of each part were terminated by hydrogen atoms. The amino acids located within a radius of 6 Å from each part of adducts were adopted. The peptide chain was divided at the C and N terminal into the individual amino acids. The ends of individual amino acids were capped with hydrogen atoms. The interaction energies of each part with individual amino acids in the InhA binding pocket were determined by using the MP2 method with the 6-31G (d) basis set. The interaction energy (IE) of adducts with each amino acid is defined as follows:

IE = E_adduct + each amino acid _– [ E_adduct_ + E_each amino acid_ ]

where E_adduct + each amino acid_ is the energy of the complex structure between adduct and each amino acid. E_adduct_ and E_each amino acid_ are the energies of adduct and each amino acid, respectively.

### 3.5. Training and test sets

The structures and activities of compounds used to set up the CoMFA and CoMSIA models are listed in Table 1. Experimental biological activities of these compounds cover 4.5 logarithmic units in term of log (1/MIC), which provides a broad and homogenous data set for CoMFA and CoMSIA studies. The data set was divided into the training set (n = 30) and the test set (n = 6) for final model development and model validation, respectively. Data classification is based on wide range of activity and structural diversity of adduct in the data set. 

### 3.6. Molecular alignment rules for CoMFA and CoMSIA modeling

The structural alignment of compounds into their bioactive conformations is essential in the set up of CoMFA and CoMSIA models. In the present study, the molecular alignment was derived from the reasonable positions of compounds in the InhA binding pocket obtained from the molecular docking calculations. The CoMFA and CoMSIA descriptor fields around the aligned compounds were generated automatically by Sybyl/CoMFA and CoMSIA routine at each lattice intersection on grid spacing of 2 Å. A sp^3^ carbon atom with a formal charge of +1 was served as the probe atom to generate CoMFA descriptors, steric (Lennard–Jones 6-12 potential) field and electrostatic (Coulombic potential) field. The probe atom was placed at each lattice point and their steric and electrostatic interactions with each atom in compounds were all calculated with CoMFA standard scaling and then compiled in a CoMFA table. The computed field energies were truncated at 30 kcal/mol for both electrostatic and steric fields. CoMSIA similarity index descriptors were derived with the same lattice box as used for the CoMFA calculations. Five CoMSIA descriptors, steric, electrostatic, hydrophobic, hydrogen bond donor and hydrogen bond acceptor, were evaluated. An attenuation factor of 0.3 was used in the CoMSIA study to generate a contour map with prominent molecular features. The partial least square (PLS) approach was employed to derive a linear relationship, in which CoMFA and CoMSIA descriptors were used as independent variables and log (1/MIC) values were used as dependent variables. To obtain the optimal number of components to be used in the final analysis, the cross-validation was performed using the leave-one-out method with a 2.0 kcal/mol column filter to minimize the influence of the noisy columns. Sequentially, a final non cross-validated analysis was performed using the optimal number of components previously identified and was then employed to analyze the results. The q^2^ or r^2^_cv_ values were used to evaluate the predictive ability of the CoMFA and CoMSIA models. 

## 4. Conclusions

Molecular docking calculations and interaction energy calculations were performed on a series of INH derivative adducts to achieve a better understanding of the crucial interactions for binding affinity of these adducts in the InhA binding pocket. The obtained results from the two approaches demonstrate that hydrogen bond interactions play an important role on the adduct binding in InhA, especially the hydrogen bonds of pyrophosphate part. To reinforce the adduct binding, the R substituents of adducts should be optimal for its cavity. Too bulky substituents generate steric effects with high repulsion energy. Conversely, too small substituents did not generate the steric effect but the preferable interactions, π-π and H-π interactions, are missing. To clearly elucidate the structural requirements of adducts that favor binding interactions in the InhA binding pocket, robust CoMFA and CoMSIA models were established. The best CoMFA and CoMSIA models include the steric and electrostatic fields suggesting that the antitubercular activity of these adducts depend on their steric and electrostatic characteristics. CoMFA and CoMSIA steric and electrostatic fields reveal that the R substituents should contain a large plane and both sides of the plane should contain an electropositive group. However, size of R substituents is controlled by the steric region above the substituent plane. Finally, the integrated results from these approaches provide valuable concepts that can be utilized for designing new and more potential effective antitubercular drugs. 
